# Monitoring of agricultural progress in rice-wheat rotation area based on UAV RGB images

**DOI:** 10.3389/fpls.2024.1502863

**Published:** 2025-01-09

**Authors:** Jianliang Wang, Chen Chen, Senpeng Huang, Hui Wang, Yuanyuan Zhao, Jiacheng Wang, Zhaosheng Yao, Chengming Sun, Tao Liu

**Affiliations:** ^1^ Jiangsu Key Laboratory of Crop Genetics and Physiology/Jiangsu Key Laboratory of Crop Cultivation and Physiology, Agricultural College of Yangzhou University, Yangzhou, China; ^2^ Jiangsu Co-Innovation Center for Modern Production Technology of Grain Crops, Yangzhou University, Yangzhou, China; ^3^ Zhenjiang Agricultural Science Research Institute of Jiangsu Hilly Area, Jurong, China; ^4^ Institute of Agricultural Sciences, Lixiahe Region in Jiangsu, Yangzhou, China

**Keywords:** UAV image, agricultural progress, deep learning, rice-wheat rotation, classification

## Abstract

Real-time monitoring of rice-wheat rotation areas is crucial for improving agricultural productivity and ensuring the overall yield of rice and wheat. However, the current monitoring methods mainly rely on manual recording and observation, leading to low monitoring efficiency. This study addresses the challenges of monitoring agricultural progress and the time-consuming and labor-intensive nature of the monitoring process. By integrating Unmanned aerial vehicle (UAV) image analysis technology and deep learning techniques, we proposed a method for precise monitoring of agricultural progress in rice-wheat rotation areas. The proposed method was initially used to extract color, texture, and convolutional features from RGB images for model construction. Then, redundant features were removed through feature correlation analysis. Additionally, activation layer features suitable for agricultural progress classification were proposed using the deep learning framework, enhancing classification accuracy. The results showed that the classification accuracies obtained by combining Color+Texture, Color+L08CON, Color+ResNet50, and Color+Texture+L08CON with the random forest model were 0.91, 0.99, 0.98, and 0.99, respectively. In contrast, the model using only color features had an accuracy of 85.3%, which is significantly lower than that of the multi-feature combination models. Color feature extraction took the shortest processing time (0.19 s) for a single image. The proposed Color+L08CON method achieved high accuracy with a processing time of 1.25 s, much faster than directly using deep learning models. This method effectively meets the need for real-time monitoring of agricultural progress.

## 1 Introduction

In the rice-wheat rotation areas, strictly following the rotation schedule is essential for the full growth and maturity of both crops and for effective agricultural progress management. Timely harvesting of rice is crucial for maximizing the utilization of seasonal and land resources for subsequent wheat planting. Late rice harvesting results in delayed wheat sowing, thereby affecting the entire growth cycle of wheat, especially its growth and maturation stages. Conversely, early harvest of rice affects its yield (Zhang L. et al., 2024). Time management and precise agricultural progress are essential in ensuring that crops are sown and harvested at optimal times, thereby improving the overall yield and quality. As rice and wheat production scales up, mastering the agricultural progress of different fields is critical for improving the overall work efficiency. A timely and accurate understanding of the agricultural progress of different fields is essential for effective agricultural management, enabling better planning and execution of key planting and harvesting activities to optimize crop production (Khormizi et al., 2024). Currently, agricultural progress monitoring is mainly conducted through manual surveys and records, which are labor-intensive and are easily susceptible to subjective factors (Du et al., 2024).

In recent years, the application of UAV remote sensing technology in agriculture has gained widespread attention. UAVs have become important tools for agricultural monitoring owing to their high flexibility, high resolution, and low cost. Additionally, UAVs can be equipped with various sensors, such as RGB, multispectral, and thermal imaging cameras, to capture high-resolution images of fields (Colomina and Molina, 2014). These sensors can be used to monitor crop growth, detect pests and diseases, and assess soil conditions (Zhang and Kovacs, 2012). In crop growth monitoring, UAV images are widely used to assess the growth and health of rice and wheat. The growth status and biomass of crops can be assessed by analyzing vegetation indices (such as normalized difference vegetation index (NDVI)) (Li et al., 2019; Najafi et al., 2023). For example, studies have shown that multispectral images obtained using UAV can accurately assess the growth status and predict the yield of rice (Zhou et al., 2017). Additionally, UAV images can be used for wheat growth monitoring and for obtaining vegetation indices from high-resolution image data to assess the growth and health of wheat (Su et al., 2019). UAV images also play an important role in pest and weed detection. The application of UAVs to identify disease spots and pest traces on crop leaves provides early warning and effective control measures in agricultural management. Previous studies have shown that multispectral images from the UAV can be used to detect rice blast disease with an accuracy of over 85% (Cao et al., 2013). The application of UAV images is highly effective in identifying common diseases in wheat, such as rust and powdery mildew (Garcia-Ruiz et al., 2013). In addition, UAV remote sensing technology has been well applied in land type classification. By obtaining high-resolution image data, researchers can classify different types of land. Previous studies have classified different land types, such as farmland, water bodies, and buildings, using multispectral images obtained using UAVs (Tong et al., 2020). In recent years, the application of deep learning technology in UAV remote sensing image processing has received considerable attention. Deep learning technology is used to develop complex neural network models for automatically extracting features from large-scale image data, enabling efficient image classification and object detection. Studies have shown that convolutional neural networks (CNNs) can significantly improve the classification accuracy of UAV images (Kamilaris and Prenafeta-Boldú, 2018). In agricultural applications, deep learning technology can achieve precise classification of crop growth, pests, and land types. For example, the deep learning model ResNet50 can be used to accurately classify different growth stages and pest conditions of rice and wheat (Fuentes et al., 2017).

Although UAV remote sensing technology has been widely used in agricultural monitoring, there are still some limitations in monitoring farming progress (Zhang W. et al., 2024). Most studies focus on crop growth status or pest and disease monitoring at a single point in time, lacking continuous monitoring of the entire farming process (Mulla, 2013)ADDIN. This shortcoming limits the comprehensive understanding of farming progress, affecting the precision of agricultural management. Traditional image processing methods lack accuracy and efficiency in complex farmland environments. Different crop types, soil conditions, and management practices across fields pose challenges for traditional methods in distinguishing similar farming activities (Timsina and Connor, 2001). For example, ploughing and harrowing are the two common farming activities with their image features, making them difficult to distinguish using traditional image processing (Li et al., 2024). Moreover, manual labeling is not only time-consuming and labor-intensive but also prone to subjective influences, resulting in poor accuracy and consistency of data. This problem is mostly common in large-scale applications, limiting the widespread use of UAV remote sensing technology in farming progress monitoring (Hunt Jr. et al., 2010).

Thus, this research utilized high-resolution image data captured by UAVs. Furthermore, these data were employed to extract image features using deep learning models, enabling precise classification of farming activities in rice-wheat rotation areas. This method improved the classification accuracy and reduced manual intervention, thereby enhancing the objectivity and consistency of the data. This study aimed 1) to identify different types of farming activities in rice-wheat rotation fields and their corresponding image features: By analyzing UAV images, image features of different farming activities, such as color, texture, and deep learning features, were extracted to achieve the classification of different farming activities; 2) to identify effective indices that can be used for farming activity classification: By comparing the classification effects of different image features, the most representative and capable indices were selected to provide data support for the subsequent construction of classification models; 3) to construct a precise and efficient farming activity classification model: Using the selected effective indices and combining them with deep learning technology, a precise and efficient farming activity classification model was developed to achieve real-time monitoring and management of farming activities in rice-wheat rotation areas. The research findings provide farmers and agricultural managers with more accurate information on plot farming activities, facilitating the optimization of agricultural management efficiency and scientific decision-making. In the context of precision agriculture management (Lu et al., 2023), this study enables real-time monitoring of farming activities, offering a scientific basis for adjusting planting and harvesting plans. This approach maximizes the utilization of land and seasonal resources, optimizes crop production systems, and enhances agricultural yield and quality. Such technological application holds not only scientific significance but also substantial practical value in advancing precision agriculture management.

## 2 Materials and methods

**Figure 1:**
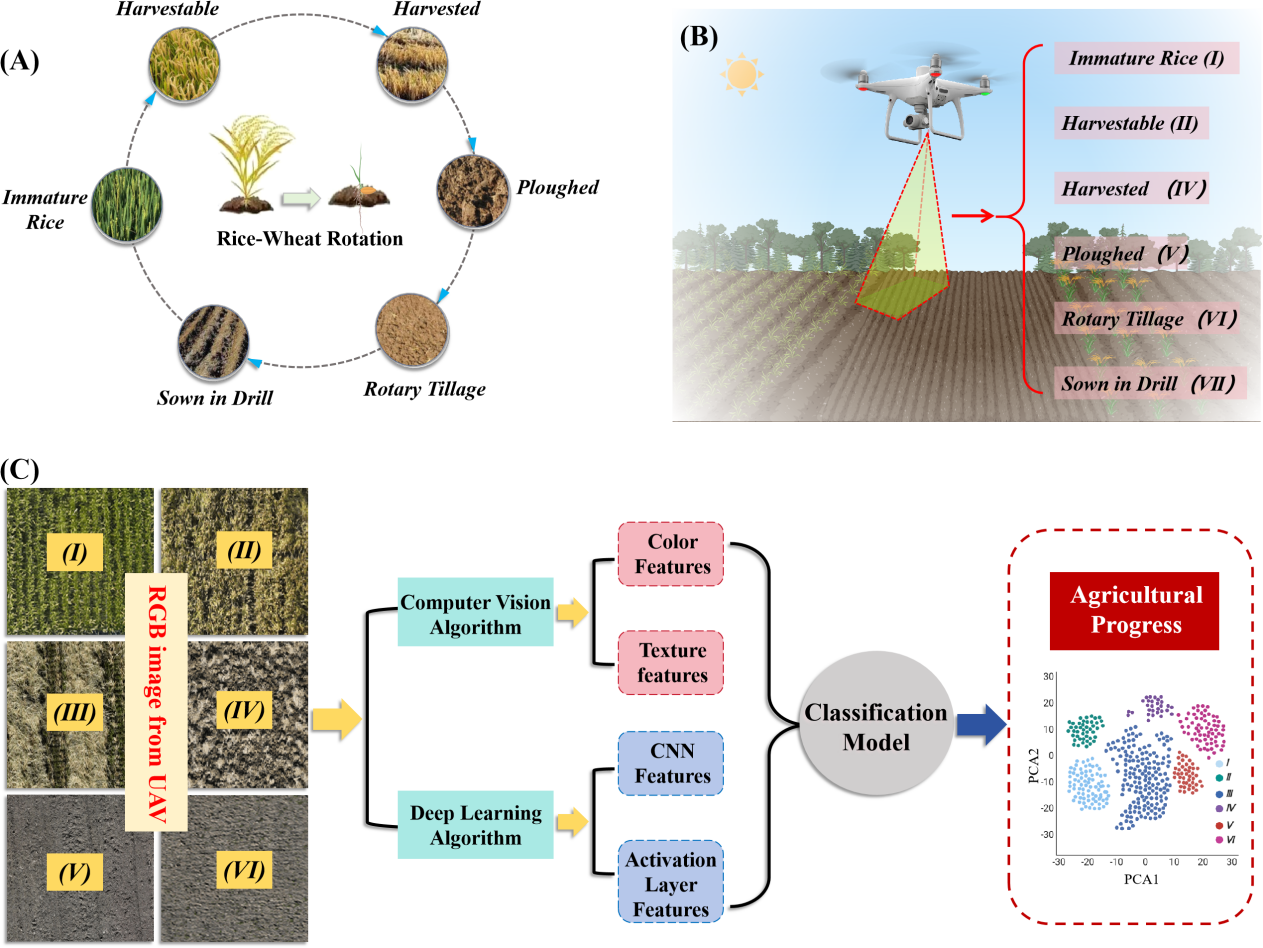
Technical flow of the study. **(A)** Depicts the six categories of agricultural operations in the rice-wheat rotation system; **(B)** offers a detailed classification of these operations: immature rice (I), harvestable rice (II), harvested rice (III), ploughed land (IV), rotary tillage land (V), and land where wheat was sown (VI); **(C)** illustrates the experimental methodology through a flowchart, comprising agricultural images acquired via RGB drones, an overview of the feature extraction algorithms, and the outputs of the classification model.

In this research, rice-wheat rotation areas were chosen as the focus, with the monitoring period spanning from the rice harvest to the completion of wheat planting. During this period, the fields were divided into six types based on farming progress: immature rice (I), harvestable rice (II), harvested rice (III), ploughed land (IV), rotary tillage land (V), and wheat was sown (VI) (**Figure 1A**). High-resolution visible light images of the fields were obtained using a UAV equipped with a high-resolution camera (**Figure 1B**). The UAV followed a predetermined route and altitude under clear and windless conditions to ensure the images covered the entire study area and maintained high quality and consistency. The obtained images were processed using computer vision and deep learning algorithms for field-type classification. First, the color features, texture features, high-level convolutional features, and activation layer features extracted using a CNN were extracted from the images, as shown in **Figure 1C**. These features were input to various classification models for field-type classification. By comparing the performance of different models, the optimal model was selected for final classification. To verify the accuracy of the classification results, ground survey data were combined to validate the UAV image classification results, ensuring consistency with the actual farming progress.

### 2.1 Experimental design and image acquisition

**Figure 2:**
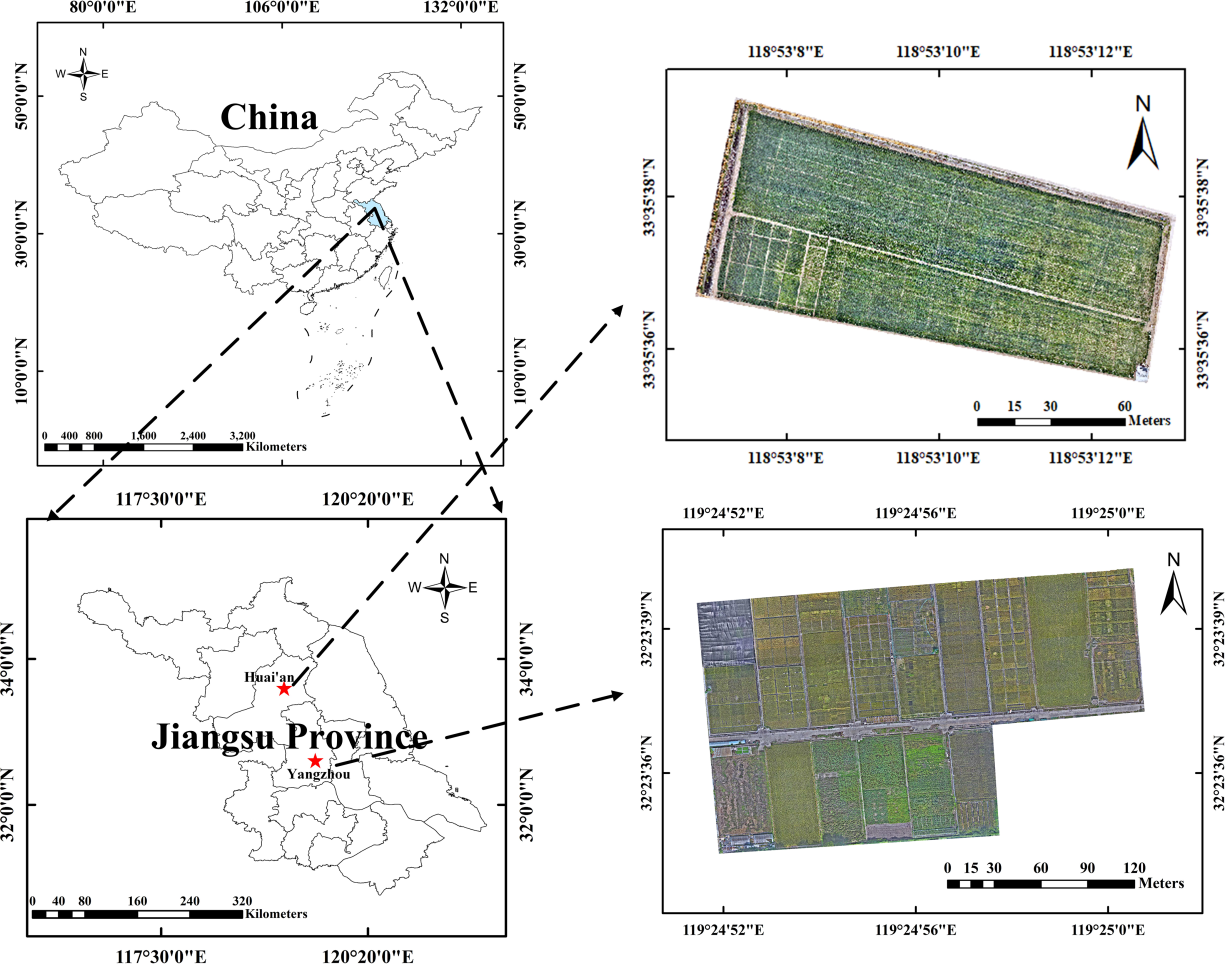
Study sites.

The experiment was conducted from 2020 to 2023 at the Modern Agricultural Science and Technology Comprehensive Demonstration Base in Huai’an City (**Figure 2**), Jiangsu Province, China (33°35′ N, 118°51′ E) and Yangzhou City (**Figure 2**), Yangzhou University Farm (32°23′ N, 119°24′ E), which belongs to the multi-year, multi-locational field experiment. The true values of the field types were obtained through surveys conducted by experienced farm staff. Determining the maturity and harvestability of the rice is challenging. The following specific criteria are used: golden yellow grains, yellow and withering leaves, drooping ears, and a rice moisture content of approximately 20% measured with a moisture meter. If these criteria are met, the rice is considered harvestable; otherwise, it is immature rice. Harvested rice refers to rice harvested using medium to large harvesters. Ploughed land refers to farmland ploughed using a ploughing tractor. Rotary tillage land refers to farmland tilled using a rotary tiller. Wheat that has been sown refers to wheat planted using a strip seeder. A DJI Mavic 3E aerial survey UAV (Shenzhen DJI Innovation Technology Co., Ltd, China) was used to collect RGB images of the fields. The visible light camera had an effective pixel count of 20 million, and the flight altitude was set to 15 m. The images were collected on clear, sunny days.

### 2.2 Image feature extraction

#### 2.2.1 Image preprocessing

In this study, the DJI Enterprise software (Shenzhen DJI Innovation Technology Co., Ltd, China) was used to complete image stitching and to obtain orthophotos. ArcMap10.8 (Esri Corporation, USA) was used to perform image alignment, geo-registration, experimental field clipping, and calibration of the UAV images. To obtain more accurate datasets based on the spatial resolution of the images, the growth conditions of the rice, and the preprocessing results of the true color images, the calibrated UAV images of the six types of fields were cut into 0.60 × 0.60 m images. During the plant target segmentation process, images smaller than 0.36 m² were excluded, resulting in a total of 30,000 0.36 m² field images. These images were manually classified into the six aforementioned datasets, with 50% of the images used for model training and 50% for model testing.

#### 2.2.2 Color indices and texture features

In this study, based on preliminary experiments (Table A.1 of Appendix A), some features that were highly correlated with the six types of fields were selected, and 12 common color vegetation indices were calculated (**Table 1**). Moreover, texture features were selected, and the contrast (CON) of UAV images was extracted using a gray-level co-occurrence matrix (Liu et al., 2024). CON reflected the image clarity and the depth of the texture grooves. The deeper the texture grooves, the greater the contrast, resulting in a clearer effect. Conversely, with a smaller contrast value, the grooves are shallow, and the effect is blurry, making it suitable for classifying different types of fields. The calculation formula is as follows:

**Table 1:** Definitions of the color indices extracted from the orthorectified RGB images.

Color indices	Formula	Reference
Excess green vegetation index (ExG)	2G-R-B	(Zhao et al., 2023)
Color intensity index (INT)	(R+G+B)/3	(Ahmad and Reid, 1996)
Kawashima index (IKAW)	(R-B)/(R+B)	ADDIN (Gitelson et al., 2002)
Visible atmospherically resistant index (VARI)	(G-R)/(G+R-B)	(Torres-Sánchez et al., 2014)
Excess red vegetation index (ExR)	1.4R-G	(Liu et al., 2020)
Green leaf index (GLI)	(2G-R-B)/(2G+R+B)	(Nie et al., 2016)
Excess green minus excess red index (ExGR)	3G-2.4R-B	(Kerkech et al., 2018)
Normalized green-red difference index (NGRDI)	(G-R)/(G+R)	(Jannoura et al., 2015)
Normalized green-blue difference index (NGBDI)	(G-B)/(G+B)	(Wang et al., 2023)
Modified green red vegetation index (MGRVI)	(G²-R²)/(G²+R²)	(Zhang et al., 2019)
Red green blue vegetation index (RGBVI)	(G²B*R)/(G²+B*R)	(Bendig et al., 2015)
Red-green ratio index (RGRI)	R/G	(Qi et al., 2021)

In the table, R, G, and B are the average color components of red, green, and blue of the true color mask image, respectively.


(1) $$\begin{array}{c} Contrast=\sum_{i}\sum_{j}{\left(i-j\right)}^{2}C_{ij} \end{array}$$

where i  and j represent the pixel values of the gray level. The value of GLCM (i,j) is the number of times that pixels with value i are adjacent to pixels with value j in the image.

#### 2.2.3 Deep learning ResNet50 features

**Figure 3:**
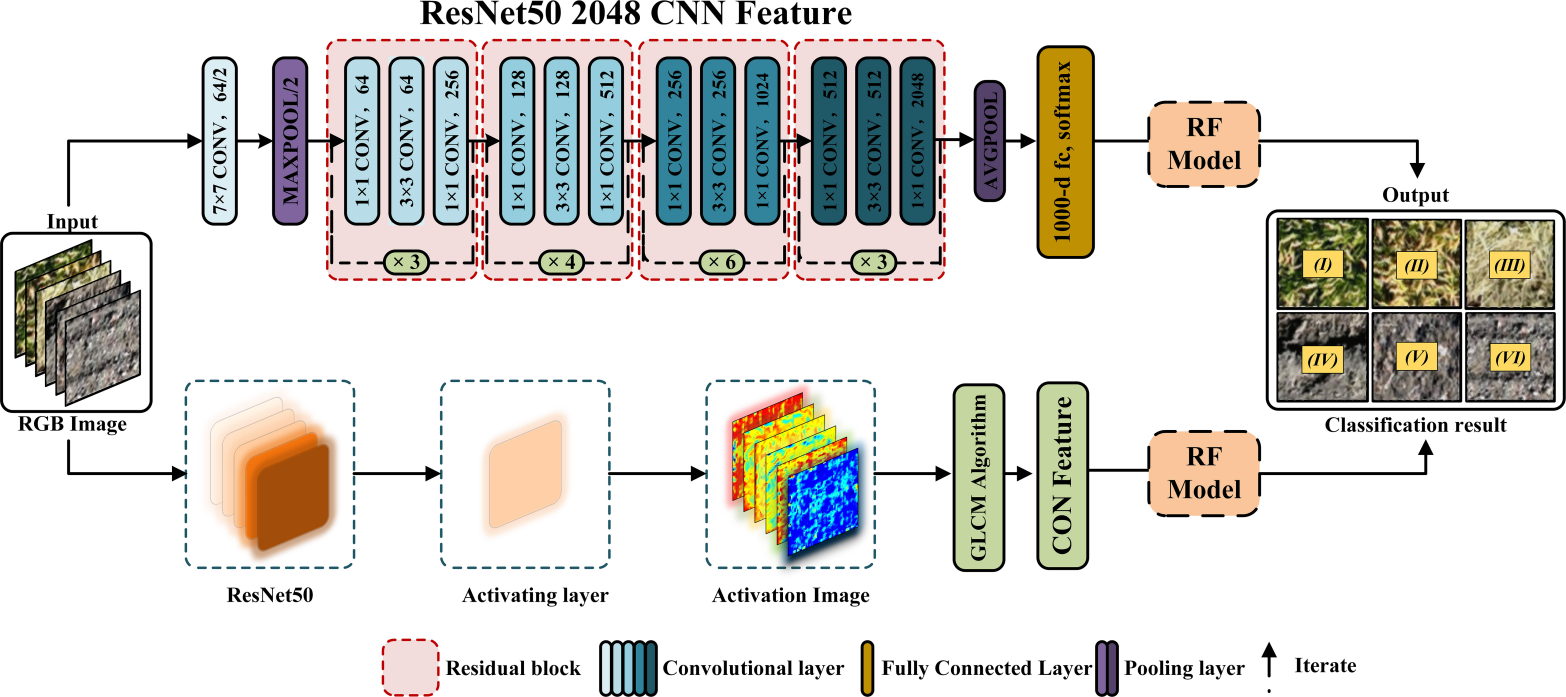
Convolutional feature extraction network architecture. The upper part of the figure provides an overview of the ResNet50 network architecture, including the algorithms involved: convolutional layers (convolution operation), pooling layers (max pooling), activation layers (ReLU activation function), fully connected layers, Softmax, and addition operations (implementing the residual structure). The lower part of the figure illustrates the process of extracting convolutional features from RGB images.

Traditional deep neural networks are prone to gradient vanishing or exploding issues when the number of layers increases, making training difficult. However, Residual Network (ResNet) can effectively solve this problem (Hu et al., 2021). The most commonly used ResNets include ResNet50 and ResNet101. Among them, ResNet50 has better recognition accuracy and real-time performance (Shabbir et al., 2021). In this study, ResNet50 was used to extract features of UAV RGB images of six types of fields and analyze and compare them with the network structure shown in **Figure 3**.

Based on the ResNet50 model, 64 activation layers were extracted, and texture feature analysis was conducted on the activation maps of each layer, as these layers reflect structural changes across six types of plots. The pre-experimental analysis methods included: 1) normalization preprocessing for each activation layer; 2) texture feature extraction using the gray-level co-occurrence matrix (GLCM) method; 3) correlation analysis to assess texture differences and classification performance across the six plot types for each activation layer; and 4) selection of activation layers that demonstrated superior texture differentiation and classification performance based on the pre-experimental findings. Six activation layers—L02, L08, L23, L36, L55, and L64—were identified in this experiment as exhibiting notable performance in texture differentiation and classification effectiveness, and their corresponding texture features were subsequently computed.

### 2.3 Modeling and validation

The random forest (RF) classification method was applied to classify six different plots. RF is a new classification algorithm proposed by the American scientist Breiman. It can efficiently handle datasets with multiple features, and it seeks the optimal solution for category attribution through cross-validation of sample features. It has advantages such as fast training speed, insensitivity to sample size, high classification accuracy, and strong noise resistance. It is one of the machine algorithms widely used in agricultural remote sensing big data intelligent learning. In the model validation stage, four metrics were used for evaluation: accuracy, recall, F1 score, and confusion matrix (Chicco and Jurman, 2020; Powers, 2020; Wen et al., 2022). The running time of models built with various methods was calculated to select the most accurate and efficient model. The formulas used are as follows:


(2) $$\begin{array}{c} Accuracy=\frac{TP+TN}{TP+FP+FN+TN} \end{array}$$

(3) $$\begin{array}{c} Recall=\frac{TP}{TP+FN} \end{array}$$

(4) $$\begin{array}{c} F1\;score=\frac{2PR}{P+R} \end{array}$$

where TP, FP, FN, and TN indicate true positive, false positive, false negative, and true negative cases, respectively. P and R represent accuracy and recall, respectively.

In this study, models were developed for six different types of plots using four different methods: color vegetation index features, color + texture vegetation index features, color + activated layer L8 features, and color + ResNet50 (2048 features). To prevent model overfitting, the dataset was divided into test sets and training sets in a 5:5 ratio. SHapley Additive exPlanation (SHAP) values were calculated for the test set. SHAP is a method used to explain machine learning model predictions. It is based on the Shapley values from game theory. It can analyze the importance of each feature in the model and quantify the contribution of each feature to the prediction of a given model for individual instances (Dikshit and Pradhan, 2021).

## 3 Results and analysis

### 3.1 Feature correlation analysis

**Figure 4:**
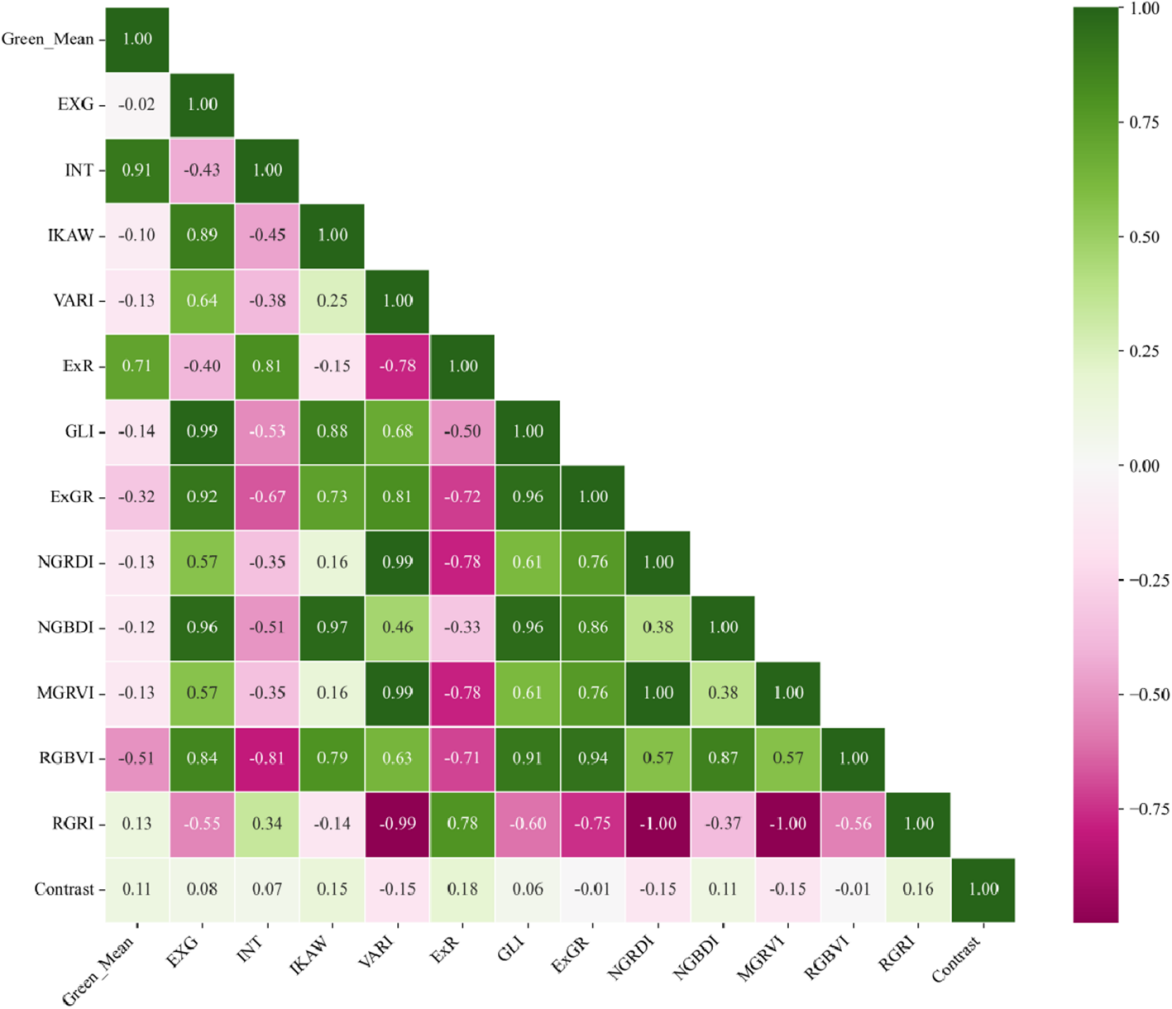
Pearson correlation analysis of RGB image features.

In the preliminary experiment (Table A.1 of Appendix A), after the image features correlated with six types of plots were initially screened, a Pearson’s correlation analysis was conducted on color and texture features commonly used in agricultural research to identify and filter out redundant features, thereby optimizing subsequent data processing and modeling work. The analysis results, shown as a heatmap (**Figure 4**), revealed a high correlation (correlation coefficient of 0.94) between EXGR and NGBDI. Additionally, the correlation coefficient between RGBVI and MGRVI was 0.94. These high correlation indicators suggest that while these features may play an important role in monitoring vegetation growth and health, they provide similar information, indicating that only one feature was retained during data simplification and model building. In subsequent analyses, a representative feature was selected from each pair of highly correlated feature groups to reduce model complexity and prevent multicollinearity issues.

### 3.2 Common image features of fields

**Figure 5:**
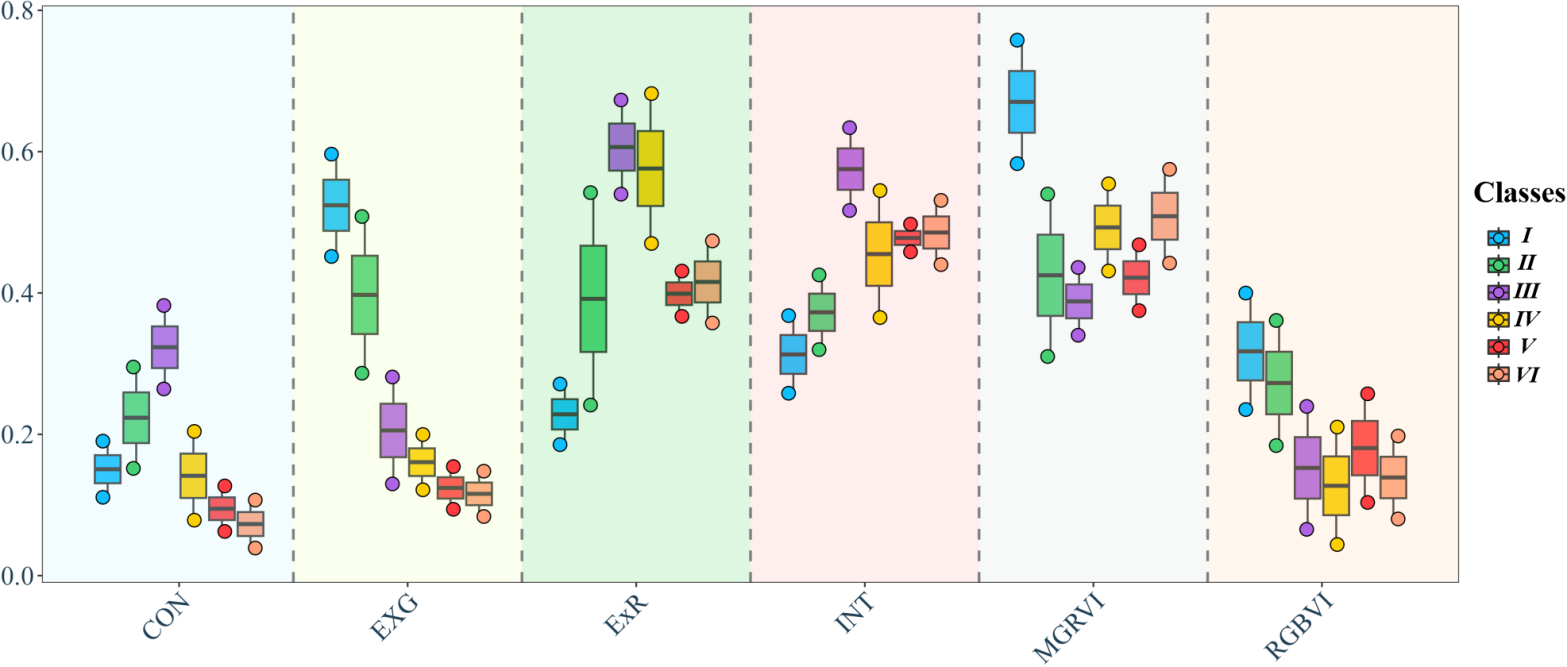
Box line plot of partial image features for six types of fields.

Analyzing the six different features of the six types of plots selected in the previous section, as shown in **Figure 5**, CON shows a significant overlap between categories V and VI. The overlap indicates that the two categories are similar in the CON feature, making CON unsuitable for distinguishing between them. However, the CON values for categories I and II were relatively dispersed. The CON values show significant differences for categories III, IV, and V. The lower CON value for category IV might help distinguish it from other categories. ExG values were significantly high in category I, clearly distinguishing it from other categories. However, there were many overlapping areas for categories IV, V, and VI, making them prone to errors when used to classify these three types of plots. ExR exhibited the opposite pattern to ExG, but the difference between categories IV and V was significant, which can be used to improve the classification of these plots. The overall performance of INT was not as good as that of the previous three features, and it also showed significant differences in categories I and II but exhibited higher overlapping areas in the latter categories. MGRVI showed high values only in category I, with varying degrees of overlap among the other five types of plots. RGBVI values for categories I and II were significantly higher than those for the other categories. However, considerable overlap was observed among the remaining four types of plots, especially between categories III and IV.

In summary, the six features show significant differences across the six types of plots. Specifically, categories I and II exhibited significant differences, making them easier to identify through color and texture features. Category III exhibited high texture features but small differences in color features compared with the other categories. Distinguishing categories IV, V, and VI was more challenging due to their small differences in both color and texture features.

### 3.3 Deep learning features

**Figure 6:**
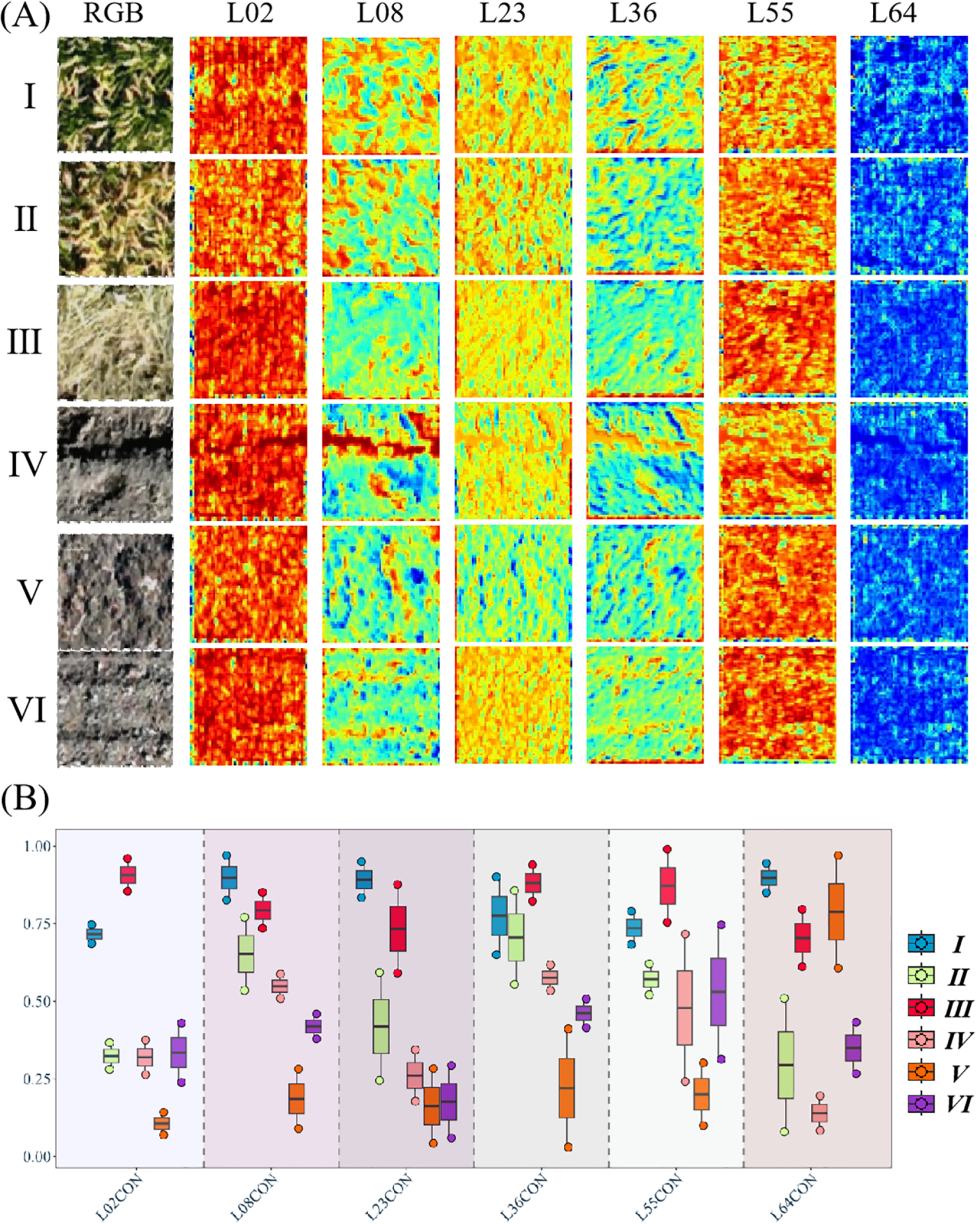
Partial RGB image of six types of fields visualized with the eighth layer of features of the activation layer. **(A)** Presents RGB images alongside six classification images derived from activation layers L02, L08, L23, L36, L55, and L64 using the ResNet50 algorithm; **(B)** demonstrates the effectiveness of contrast features from the activation layers in field classification applications.

To further improve the accuracy of plot classification and enhance the classification accuracy of plots with similar color and texture features, we used ResNet50 to extract convolution features (mean and variance of features extracted by convolutional networks) of images from six types of plots. It was used to analyze the 64 activation layers of ResNet50, selecting L02, L08, L23, L36, L55, and L64 activation layers with significant differences among the six types of plots for further analysis and screening. The activation layers of the six types of plots are shown in **Figure 6A**. The activation layer images clearly distinguish changes in the surface structure of the plots, which is beneficial for plot differentiation. Further analysis of the contrast of the six activation layer images showed that the activation layer features of the six types of plots had significant differences compared with their color and texture features. As shown in **Figure 6B**, except for L23CON, significant differences were observed in the activation layer features of category V and VI plots. The observed difference can mitigate the difficulty in classifying these two types of plots using color and texture. Additionally, slight differences were observed among categories II, IV, V, and VI in L02CON, with some overlap with category IV. Moreover, slight differences were observed between categories I and II in L36CON. L55CON was similar to L02CON, with slight differences observed between categories V and VI, but with a larger overlapping than L02CON. L64CON exhibited overall differences among the six types of plots, with some overlap observed only between categories III and V and categories II and VI. In L08CON, differences were observed among the six types of plots with minimal overlapping values, except for small overlaps between categories II, III, and IV. Therefore, this feature was selected to establish the plot classification model.

### 3.4 Principal component analysis

**Figure 7:**
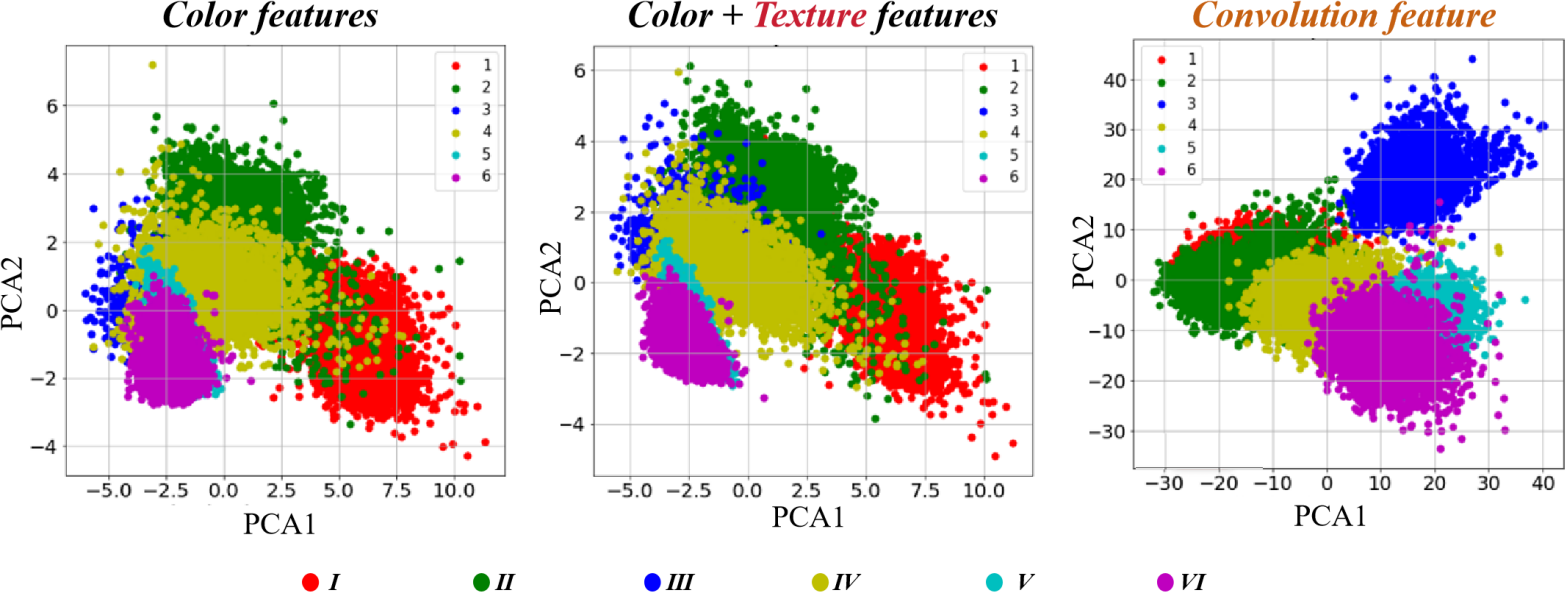
PCA plot of features.

Principal Component Analysis (PCA) was conducted using color indices, combined color and texture indices, and convolutional features to examine the differences among data groups across six field categories (**Figure 7**). For the PCA based on color features, there was limited overlap between category I and the other categories, whereas significant overlap was observed among the remaining categories. This suggests that individual color indices exhibit limited discriminatory power. Additionally, the variance explained by the first two principal components was below 70%, indicating that the information is distributed across multiple components. When texture features were incorporated, the PCA results demonstrated improved category separation compared to those based solely on color features. Specifically, the boundary between category I and category IV became more distinct, and categories V and VI exhibited clearer clustering patterns. These improvements highlight the substantial contribution of texture features to the principal components, with the explained variance exceeding 80%. In contrast, convolutional features extracted using ResNet50 displayed a different pattern compared to color and texture features. Category III fields were distinctly separated from the others, and the boundary between categories V and VI was well-defined. However, performance for categories I and II was comparatively weaker. Notably, ResNet50 achieved an explained variance of 90–95%, reflecting the high concentration of inter-category differences in the low-dimensional space. The PCA findings further suggest that while color features are effective for rapid preliminary classification, auxiliary features may be necessary to distinguish complex or highly similar categories. Texture features contributed additional spatial information, enhancing the separation of categories with similar colors. Meanwhile, convolutional features provided information distinct from both color and texture, enabling effective differentiation among categories.

### 3.5 Image feature classification results

**Figure 8:**
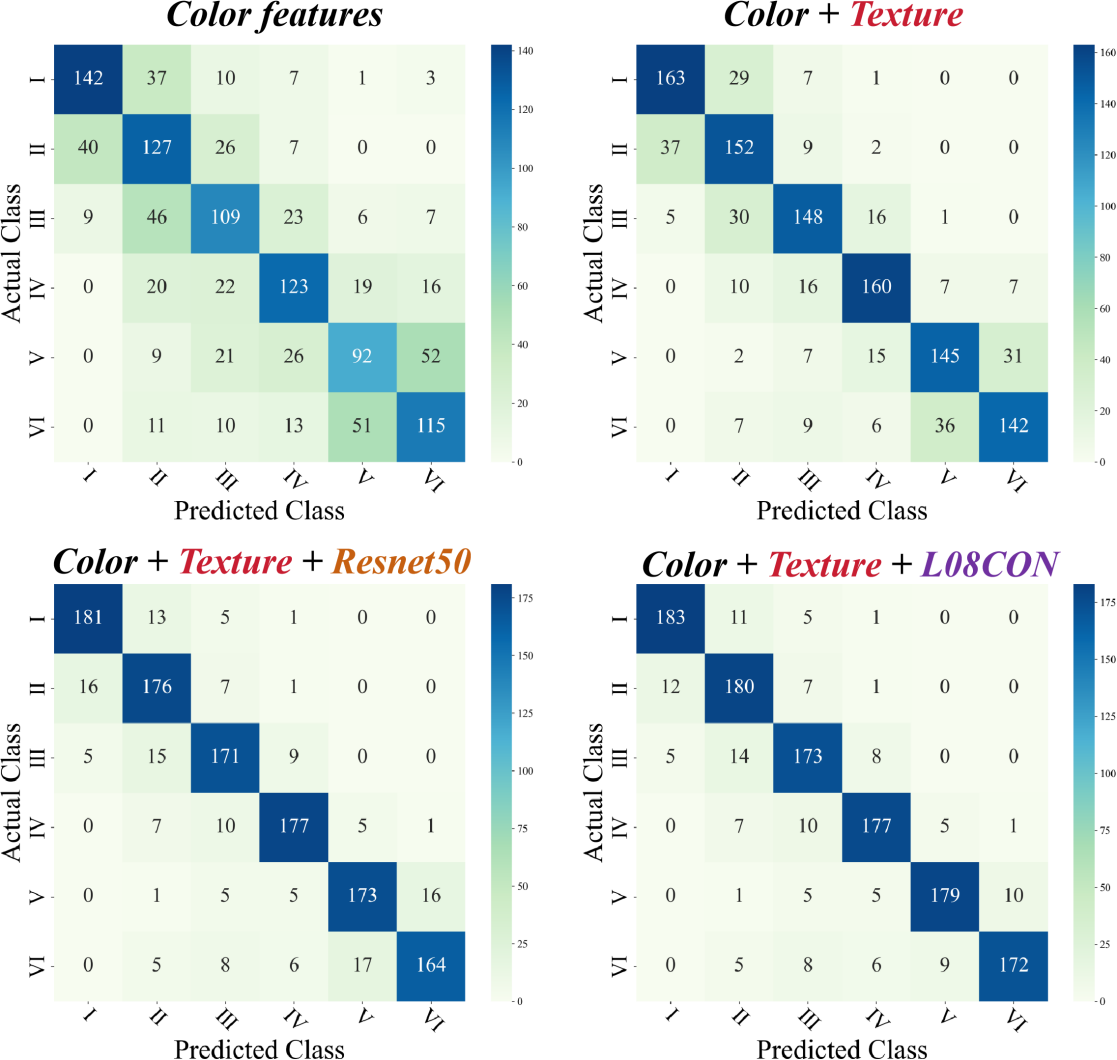
Four methods of confusion matrices.

An RF classification model and different feature combinations were used to classify the plots. The confusion matrix is shown in **Figure 8**. When only color features were used for classification, both the overall misidentification and omission values were high, with significant errors. When combining color and texture, the recognition accuracy improved, especially in reducing the misidentification and omission of category V plots. The introduction of ResNet50 features (2048 sets) or L08CON features significantly improved accuracy, particularly in addressing the misidentification and omission issues between category V and VI plots, with L08CON performing better than ResNet50.

Further analysis of the overall accuracy, recall, F1 score, and run time for different combinations, as shown in **Table 2**, reveals that the overall accuracy is consistent with the confusion matrix analysis results. Both Color+L08CON and Color+ResNet50 achieved an accuracy of over 98%. The addition of texture on the basis of Color+L08CON did not significantly improve classification accuracy. In terms of classification time per image, the color feature extraction time was the shortest, at 0.19 s. The ResNet50 feature extraction time was the longest, exceeding 6 s; the texture feature extraction time was relatively long, exceeding 4 s. The L08CON extraction time was approximately 1.20 s. Although the Color+Texture+L08CON combination achieved the highest accuracy, its feature extraction time was relatively long, which is not conducive to real-time plot detection.

**Table 2:** Classification results and running time of each methodological model.

Validation index	Model				
	Color features	Color+ Texture	Color+ L08CON	Color+ ResNet50	Color+Texture+ L08CON
Accuracy (%)	80.54%	90.63%	98.76%	98.04%	98.96%
Recall (%)	82.01%	91.28%	98.17%	98.37%	98.66%
F1 score (%)	79.92%	91.21%	98.54%	98.19%	98.62%
Run time (s)	0.19	4.71	1.25	6.93	6.35

**Figure 9:**
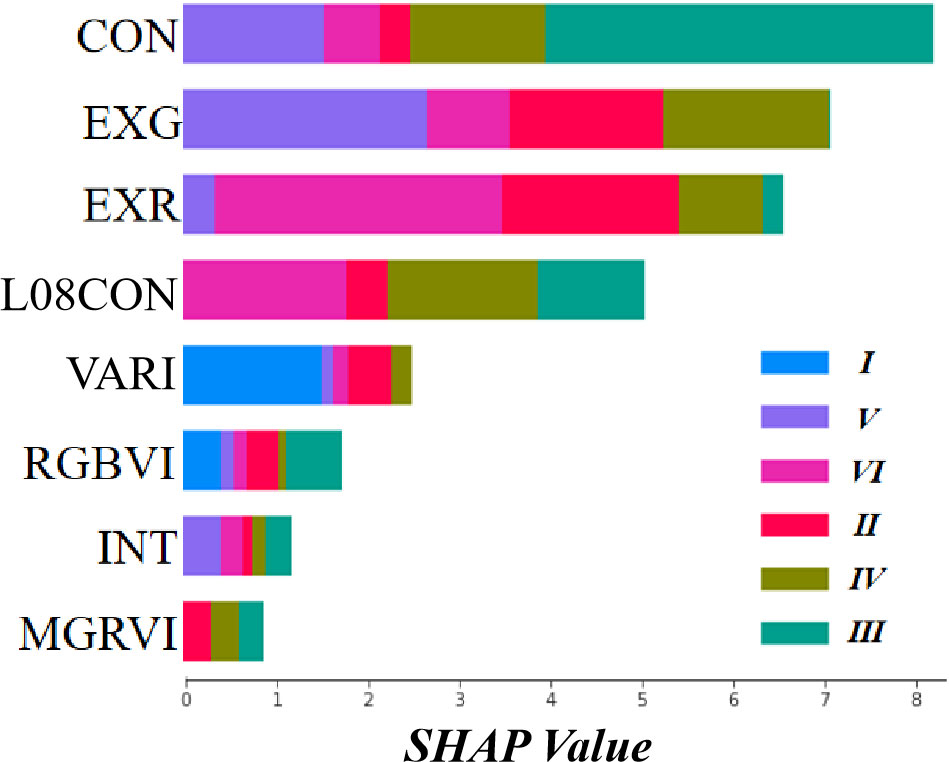
Visualization of SHAP values for each group of features. In the legend, (I)–(III) represent six different plot types: immature rice (I), harvestable rice (II), harvested rice (III), ploughed land (IV), rotary tillage land (V), and wheat has been sown (VI). The colored rectangles indicate their respective contribution proportions in the model, with longer rectangle lengths signifying greater contributions.

The test set for the RF classification model, built with four methods, was calculated to obtain the SHAP values of each feature in the model and visualize them (**Figure 9**). The SHAP values reveal that the texture feature CON, the color features ExG and ExR, and L08CON contribute the most to the model. The features VARI, RGBVI, INT, and MGRVI have the least impact, while the contributions of the remaining features are even smaller and not listed here.

## 4 Discussion

This research integrated UAV imaging technology with diverse feature extraction and classification methods to achieve precise monitoring of agricultural activities in rice-wheat rotation areas. The results showed that different image features have unique strengths and limitations in field classification. Initially, the feature correlation analysis demonstrated that several color and texture features (e.g., EXGR and NGBDI, RGBVI and MGRVI) showed strong correlations. This observation implies that redundant features were excluded during data simplification and modeling processes to reduce the complexity of the model, aligning with methodologies reported in previous studies (Mutlag et al., 2020; Xie and Yang, 2020). The retention of only one representative feature after screening improved the computational efficiency and the stability of the model. In analyzing common color vegetation index features in fields, although color features showed significant differences between certain categories, their effectiveness was limited in distinguishing similar categories (e.g., IV, V, and VI). These results reveal the issue that solely relying on a single type of feature in field classification may lead to classification inaccuracy, especially for post-rice harvest field images. To address this issue, in this study, we further introduced deep learning features. The convolutional features and activation layer features extracted with ResNet50 significantly improved the classification accuracy, especially in distinguishing categories with similar color and texture features (e.g., V and VI). The results showed that convolutional features had significant advantages in capturing surface structure changes in fields, compensating for the shortcomings of traditional color and texture features in distinguishing certain categories. PCA further confirmed the effectiveness of feature combinations. Although color features are suitable for rapid preliminary classification, texture features and convolutional features are needed to distinguish complex or similar categories. Convolutional features provided complementary information to color and texture features in classification, effectively enhancing the separation of different categories. The final classification results showed that the integrated model using color, texture, and convolutional features (e.g., Color+L08CON and Color+ResNet50) achieved an accuracy of over 98%, significantly higher than the classification results of single features. Although the Color+Texture+L08CON combination achieved the highest accuracy, its feature extraction time was relatively long, making it unsuitable for real-time field detection. Therefore, in practical applications, a balance must be established between classification accuracy and processing time.

**Figure 10:**
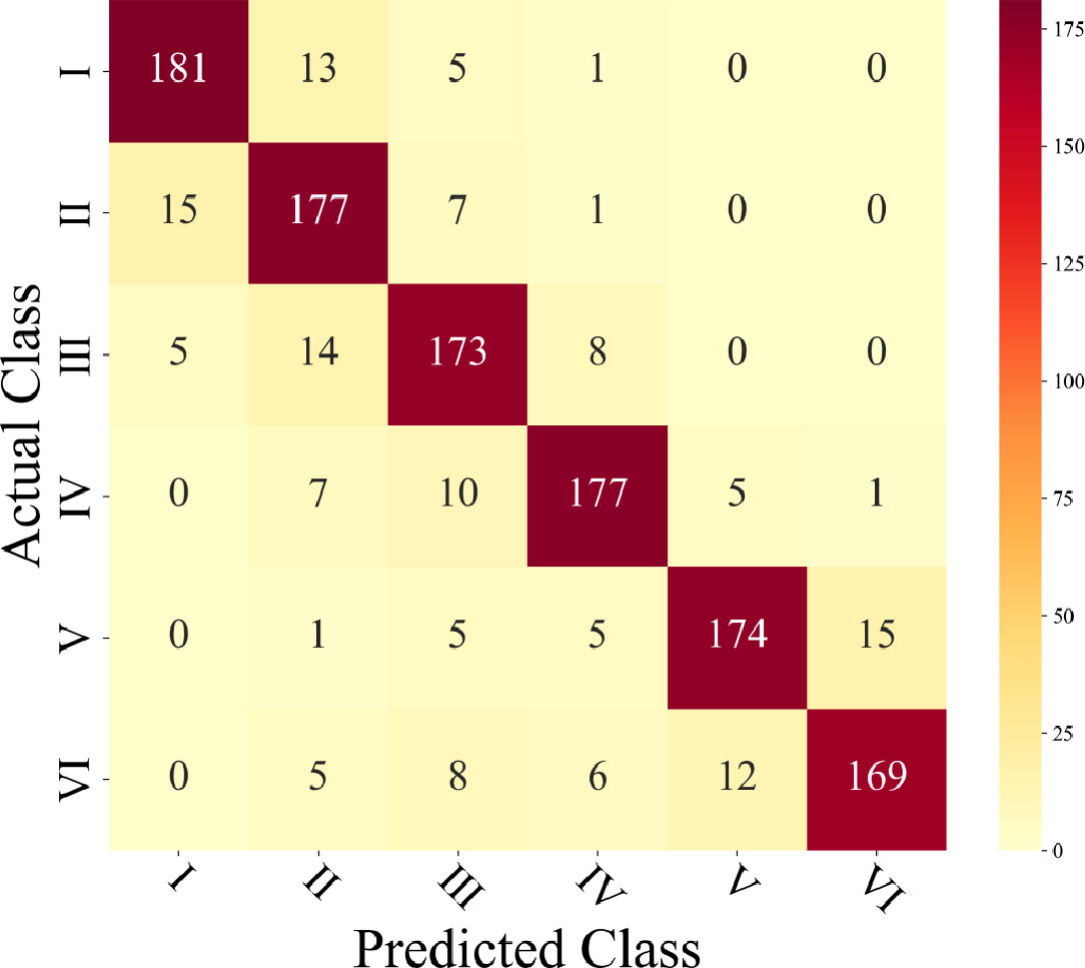
Confusion matrix for LeNet-5 results.

This study evaluated and compared the classification performance of the widely used deep learning algorithm LeNet-5 on field plots (**Figure 10**). The findings revealed that, following extensive training, LeNet-5 demonstrated satisfactory classification accuracy, particularly for categories II, III, and IV, yielding results comparable to those of the method proposed in this study. However, for categories I and V, LeNet-5 exhibited significantly lower accuracy compared to the method introduced here. Deep learning models such as LeNet-5 demand extensive initial training, with their accuracy being heavily dependent on the diversity and comprehensiveness of the dataset. Consequently, achieving high-precision classification necessitates large and diverse training datasets, which can present practical challenges in terms of data collection and annotation. Furthermore, in terms of processing speed, LeNet-5 required approximately 3 seconds longer per image than the method employed in this study, potentially limiting its applicability for real-time detection in large-scale field monitoring. While LeNet-5 performed well in identifying certain categories, its elevated training and runtime requirements pose challenges. In contrast, the method proposed in this study offers a more balanced and efficient approach, optimizing classification performance, training effort, and runtime efficiency. Therefore, for practical applications, the method presented here ensures robust classification accuracy while maintaining superior real-time performance and operational feasibility. Future research could focus on further optimizing deep learning models and exploring their integration into agricultural progress monitoring within rice-wheat rotation systems. This effort could involve combining deep learning approaches with traditional image processing techniques to achieve enhanced efficiency in agricultural monitoring.

The method used in this study has similarities and some significant differences with traditional remote sensing technology in land type classification research. The proposed agricultural progress classification method relies on high-resolution image data and uses various image features for classification. Remote sensing technology extracts spectral, texture, and shape features of land cover types from satellite images or UAV images and then uses classification algorithms to classify different land types (Al-Najjar et al., 2019), which is similar to the method used in this study. Meanwhile, the proposed method also extracts color features, texture features, and convolutional features. Additionally, it uses an RF classification model for field classification. These methods essentially distinguish different land cover types by analyzing the features of image data (Bai et al., 2021). However, due to the tight timing of agricultural progress and the slight differences in arable land types, using only traditional remote sensing classification methods is not ideal for agricultural progress classification. This study mainly focused on real-time monitoring of agricultural progress, not just the static classification of land cover types. Traditional remote sensing research is mostly used for large-scale land use and cover change monitoring, with low temporal resolution (Wang et al., 2022). In contrast, this study used UAV images to obtain high-frequency image data, enabling high temporal resolution monitoring and timely management of agricultural activities. A significant feature of this study is the application of convolutional features. The convolutional features extracted with deep learning models (such as ResNet50) proved more effective in distinguishing subtle changes under the same land cover types. This efficacy enabled our study to accurately differentiate subtle agricultural changes, such as differences in ploughing, tilling, and seeding land types, where traditional remote sensing classification methods are limited. Convolutional features provide richer spatial information, facilitating the capture of small changes in field surface structures, thus improving classification accuracy. The study inherits some classic methods of remote sensing technology in land classification but enhances real-time performance and classification accuracy by introducing UAV and deep learning technology, making it particularly suitable for agricultural progress monitoring in rice-wheat rotation areas.

## 5 Conclusion

This study successfully achieved precise monitoring of agricultural progress in rice-wheat rotation areas by integrating UAV imaging technology with various feature extraction and classification methods. The findings demonstrate that multiple image features offer distinct advantages in plot classification. By combining color, texture, and convolutional features extracted through deep learning, significant improvements in classification accuracy were achieved. The results indicate that integrated models using color, texture, and convolutional features (such as Color+L08CON and Color+ResNet50) can achieve an accuracy exceeding 98%, significantly reducing overall misclassification and omission rates compared to methods relying on a single feature. Specifically, the Color+L08CON model attained an accuracy of 98.76%, while the model using only color features achieved an accuracy of 80.54%. In terms of processing time for a single image, color feature extraction was the fastest at 0.19 seconds, followed by Color+L08CON at 1.25 seconds, whereas ResNet50 feature extraction took the longest, exceeding 6 seconds. The proposed Color+L08CON model not only achieved high accuracy but also minimized the processing time per image, meeting the requirements for real-time land type detection. Overall, this study demonstrated that combining UAV imaging with multiple feature extraction and classification methods enables efficient and accurate monitoring of agricultural progress in rice-wheat rotation areas. By adjusting model parameters and expanding training datasets, this method can be adapted to complex field environments and diverse crop planting patterns, offering reliable technological support for precision agricultural management. Future research should focus on optimizing feature extraction and classification algorithms to enhance real-time monitoring efficiency and accuracy. Additionally, integrating other remote sensing data and ground sensors would enable the development of a more comprehensive monitoring system, supporting scientific agricultural management and sustainable development.

## Data Availability

The raw data supporting the conclusions of this article will be made available by the authors, without undue reservation.
