# Supplementary material for: Monitoring of agricultural progress in rice-wheat rotation area based on UAV RGB images
Source: Front Plant Sci. 2025 Jan 9;15:1502863. doi: 10.3389/fpls.2024.1502863 (PMC11754401; doi:10.3389/fpls.2024.1502863)
Supplement: Supplementary file 1 [file Table1.docx]

**Table A.1** Correlation of texture features in RGB images.

| **Feature name** | **Correlation coefficient (r)** | **Contribution (%)** | **Redundancy assessment** |
| --- | --- | --- | --- |
| Contrast | 0.85 | 52.7 | Low redundancy |
| Correlation | 0.62 | 25.3 | Moderate redundancy |
| Energy | 0.51 | 12.5 | Moderate redundancy |
| Homogeneity | 0.44 | 10.2 | High redundancy |
| Entropy | 0.43 | 9.3 | High redundancy |

**Note:** Contrast abbreviated in the manuscript as CON.
